# The Influence of Citrus Pectin and Polyacrylamide Modified with Plant-Derived Additives on the Properties of α-TCP-Based Bone Cements

**DOI:** 10.3390/polym16121711

**Published:** 2024-06-15

**Authors:** Joanna P. Czechowska, Piotr Pańtak, Kinga J. Kowalska, Jeevitha Vedaiyan, Mareeswari Balasubramanian, Sundara Moorthi Ganesan, Konrad Kwiecień, Elżbieta Pamuła, Ravichandran Kandaswamy, Aneta Zima

**Affiliations:** 1 Department of Ceramics and Refractories, Faculty of Materials Science and Ceramics, AGH University of Krakow, Al. Mickiewicza 30, 30-059 Kraków, Polandkjkowalska@agh.edu.pl (K.J.K.); 2 Department of Rubber and Plastics Technology, Madras Institute of Technology Campus, Anna University, Chromepet, Chennai 600 044, Indiavenibala18@gmail.com (M.B.); sundaramoorthi1997@gmail.com (S.M.G.); 3 Department of Biomaterials and Composites, Faculty of Materials Science and Ceramics, AGH University of Krakow, Al. Mickiewicza 30, 30-059 Kraków, Poland

**Keywords:** α-tricalcium phosphate, bone cements, polyacrylamide, sago starch, neem, rambutan

## Abstract

Materials based on highly reactive α-tricalcium phosphate (α-TCP) powder were developed and evaluated. Furthermore, the impact of different polymeric additives, such as citrus pectin or polyacrylamide (PAAM) modified with sago starch, neem flower, or rambutan peel, on the physiochemical and biological properties of the developed materials was assessed. The addition of modified PAAM shortened the setting process of bone cements and decreased their compressive strength. On the other hand, the addition of citrus pectin significantly enhanced the mechanical strength of the material from 4.46 to 7.15 MPa. The improved mechanical properties of the bone cement containing citrus pectin were attributed to the better homogenization of cementitious pastes and pectin cross-linking by Ca^2+^ ions. In vitro tests performed on L929 cells showed that 10% extracts from α-TCP cements modified with pectin are more cytocompatible than control cements without any additives. Cements containing PAAM with plant-derived modifiers show some degree of cytotoxicity for the highly concentrated 10% extracts, but for diluted extracts, cytotoxicity was reduced, as shown by a resazurin reduction test and live/dead staining. All the developed bone substitutes exhibited in vitro bioactivity, making them promising candidates for further biological studies. This research underscores the advantageous properties of the obtained biomaterials and paves the way for subsequent more advanced in vitro and in vivo investigations.

## 1. Introduction

Calcium phosphate cements (CPCs) are self-setting bone substitutes widely used in various orthopedic applications thanks to their biocompatibility, osteoconductivity, and resorbability [1]. Their ability to integrate with biological tissues, encourage bone formation, and undergo gradual resorption aligns with the dynamic nature of the human body’s regenerative processes [2]. Recent studies explored CPC formulations containing alpha-tricalcium phosphate (α-TCP), which undergoes hydrolysis to non-stoichiometric hydroxyapatite when mixed with water-based solutions [3].

However, despite these advantageous attributes, some challenges still exist in the development and practical application of α-TCP-based bone cements. Specifically, improvements are needed in their setting times, rheological properties, and mechanical strength [4].

**Figure 1:**
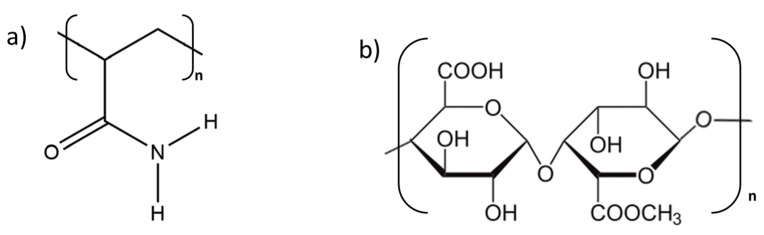
Chemical structure of (**a**) polyacrylamide (PAAM) and (**b**) pectin (chemical structure containing both galacturonic acid and its methoxylated units).

A common approach to enhance the physicochemical properties of CPCs involves adding specific types of polymers to the formulations of bone cements. Among these, polyacrylamide (PAAM) and citrus pectin stand out as promising options due to their unique properties. Polyacrylamide is a water-soluble synthetic polymer known for its impressive mechanical properties, strong adhesion, proper hygroscopicity, high hydrophilicity, and non-toxic nature [5]. The amine groups in acrylamide monomers have two hydrogen atoms, enabling them to form hydrogen bonds with functional groups that have available lone pairs of electrons (Figure 1a) [6]. Unfortunately, PAAM is biologically inert, and modification is necessary to improve its biological properties. The most common approaches found in the literature involve combining PAAM with bioactive calcium phosphates (i.e., hydroxyapatite) or antibacterial agents [7,8,9]. Various approaches have been used to obtain bioactive composites. For instance, Fang et al. [8] developed arobust, osteoconductive hydrogel through a single-step copolymerization of acrylamide and urethacrylate dextran, which was followed by hydroxyapatite mineralization. Wang et al. [9] developed a composite coating for endotracheal tubes containing PAAM, gelatine, and silver nanoparticles that prevents occlusion and biofilm-related infections. The developed coating, with dual antibacterial and antifouling effects, exhibited strong resistance against *Staphylococcus aureus* and *Pseudomonas aeruginosa* as well as favorable biocompatibility. In addition to improving the biological properties of composite materials containing PAAM, it is also possible to use PAAM hydrogels to enhance their physiochemical characteristics. Xia et al. [10] investigated the effect of calcium ions on the performance of polyacrylamide membranes. The presence of Ca^2+^ ions improved the membranes’ performance due to the occurrence of electrostatic interactions between the carboxyl groups of the polymer and calcium cations. By combining materials containing calcium ions, such as α-TCP, with polyacrylamide modified with plant-derived additives, it is possible to obtain composites with unique properties. Citrus pectin (CP) is a natural polymer derived from citrus fruits that has been recently used as a component of calcium phosphate-based bone cement formulations. The pectin structure strongly depends on the pectin source and extraction conditions. The homogalacturonan component of pectin is made up of galacturonic acid residues linked in chains by α-(1-4) glycosidic bonds. The carboxyl groups of these galacturonic acids are partially esterified, and the degree of esterification is a crucial chemical parameter of pectin (Figure 1b) [11].

Beyond its potential to increase the strength of bone cements, CP has demonstrated the ability to improve the injectability of cement pastes [12] and can positively influence the biological properties of biomaterials. For example, Munarin et al. [13] investigated the use of pectin, modified with or without an RGD-containing oligopeptide, as an extracellular matrix alternative for immobilizing MC3T3-E1 preosteoblasts. The study revealed that pectin may be potentially used as an injectable cell vehicle for bone tissue regeneration, showing enhanced cell adhesion and proliferation. On the other hand, citrus pectin, as a polyanionic polymer can interact with calcium phosphate ions, causing an enhancement of the materials’ strength due to the presence of electrostatic interactions [14].

Recently, a lot of attention has been paid to molecules extracted from plants, which have been used in traditional medicines. Neem (*Azadirachta indica*) shows potential because of its abundance in antioxidants and other valuable active compounds such as azadirachtin, nimbolinin, nimbin, nimbidin, nimbidol, salannin, and quercetin [15]. Rambutan (*Nephelium lappaceum*) is high in antioxidants, dietary fibers, vitamins, minerals, and bioactive compounds [16]. Thus, these plant-derived compounds seem to be valuable modifiers of polymers intend for medical applications, such as PAAM. Another compound that has been focused on is Sago (*Metroxylon sagu*) starch, which, apart from its nutritional properties, can be used to modify the properties of different polymers. It has been shown that polyacrylamide-based hydrogels with Sago starch, in addition to gaining nutritional properties, also possess increased hydrophilicity and improved biodegradability.

The aim of this study was to develop and study the cementitious-type biomaterials based on α-TCP and to determine the effect of various polymeric additives, containing plant-derived ingredients, introduced in the powder phase on the physicochemical and biological properties of those materials. Bone substitutes composed of highly reactiveα-TCP as the setting phase and citrus pectin or PAAM modified with sago starch, neem peel, or rambutan peel were obtained and investigated. Based on our knowledge, these studies mark a pioneering exploration into the effects of polyacrylamide (PAAM) modified with plant-derived substances on cementitious materials based on α-TCP. They serve as a foundational platform for further research in bone tissue engineering and showcase novel, potentially advantageous applications of natural polymers as modifiers for bioceramic bone cements.

## 2. Materials and Methods

### 2.1. Materials

#### 2.1.1. Synthesis of α-TCP

The initial α-tricalcium phosphate (α-TCP) powder was synthesized by the wet chemical method described previously [17]. As reagents, Ca(OH)_2_ (≥99.5%, POCH, Gliwice, Poland) and H_3_PO_4_ (85.0%, POCH, Gliwice, Poland) were applied. In summary, following synthesis, the α-TCP precipitate underwent drying, sintering at temperatures exceeding 1250 °C (for 5 h), grinding in an attritor mill (for 4 h), and sieving to achieve a particle size below 63 µm.

#### 2.1.2. Synthesis of Polyacrylamide Hydrogels (PAAMs)

Polyacrylamide hydrogels (PAAMs) were synthetized according to the previously described method [18]. Briefly, acrylamide monomer (AAM) (1.0 g) and N,N’methylenebisacrylamide (MBAAM) (chemical grade, BASF, Ludwigshafen am Rhein, Germany) (1.0 wt.%) were dissolved in 50 mL of deionized water, and 2 mL of 1.0 wt.% potassium sulfate solution was added. The solution was flushed with nitrogen for 20 min to remove dissolved oxygen. Polymerization was carried out in a constant temperature bath at 55 °C for 30 min. The polyacrylamide gel underwent a washing process with distilled water to eliminate any remaining unreacted monomer and residual initiator. This was achieved by immersing the gel in deionized water for 24 h. The yield of the cross-linked gel was found to be 97.0%.

#### 2.1.3. Synthesis of Sago Starch/Neem Flower/Rambutan Peel-Modified Polyacrylamide Hydrogels

Sago starch/neem flower/rambutan peel modified polyacrylamide hydrogels were synthetized according to the modification of the previously described method [18]. The acrylamide monomer (AAM) (1.0 g) and N,N’methylenebisacrylamide (MBAAM) (chemical grade, BASF, Ludwigshafen am Rhein, Germany) (1.0 wt.%) and 5.0 wt.% of sago starch/neem flower/rambutan peel with respect to an acrylamide monomer were dissolved in 50 mL of deionized water, and 2 mL of 1 wt.% potassium sulfate solution was added. The solution was flushed with nitrogen for 20 min to remove dissolved oxygen. The polymerization process took place in a consistent temperature bath at 55 °C for 30 min. The cross-linked starch-grafted polyacrylamide gel underwent a washing step with distilled water to eliminate any leftover monomer and residual initiator. The washing was accomplished by immersing the gel in deionized water for 24 h. Subsequently, the gel was dried at 40 °C. Before the samples’ preparation, hydrogels were grinded and sieved to achieve a particle size below 63 µm.

#### 2.1.4. Bone Cements Preparation

Five types of materials were prepared by mixing the solid phase α-TCP and polymeric additives (3.0 wt.%) with the liquid phase. The liquid phase of the developed materials consisted of a 2 wt.% aqueous solution of disodium phosphate, which is commonly used as a setting accelerator in calcium phosphate-based cementitious materials. Additionally, for the comparison of synthesized hydrogels with other similar polymers, one material was modified by incorporating powdered citrus pectin (low esterified amidated pectin from citrus peels; degree of esterification—27.4%, degree of amidation—22.8%, Herbstreith & Fox, Werder (Havel), Germany). To obtain cementitious pastes, the homogenized components of the solid phase were mixed with the liquid phase for approximately 30 s. The initial composition of the prepared bone cements, as well as a liquid to powder ratio (L/P), are presented in Table 1.

### 2.2. Methods

#### 2.2.1. Phase Composition

The X-ray diffraction (XRD) analysis was carried out using Cu Kα radiation (1.54 Å) at 30 kV and 10 mA. The examination encompassed the 2θ range of 10–40° at 0.04° intervals, employing a scanning speed of 2.5°/min with a D2 Phaser diffractometer (Bruker, Ballerica, MA, USA). The range of 2 theta angles was within the range 10–40 degrees due to the absence of significant reflexes originating from calcium phosphates above 40 degrees. Limiting the range of observed angles allowed for the precise observation and analysis of reflexes originating from calcium phosphates. The diffractograms obtained were compared with the International Centre for Diffraction Data for α-tricalcium phosphate (α-TCP; 00-009-0348) and hydroxyapatite (HA; 01-076-0694) to identify crystalline phases. Phase quantification was performed using TOPAS software (Version 4.2.0.1, 2011, Bruker, Ballerica, MA, USA) through Rietveld refinement. Each measurement was conducted in triplicate, and the results are presented as mean ± standard deviation (SD).

#### 2.2.2. Setting Times

The setting times were measured in accordance with the ASTM C266-20 standard, using Gilmore Needles (Humbold MFG Co., Norridge, IL, USA) [19]. The Gillmore Apparatus, comprising two steel-weighted needles, was employed for this purpose. The initial setting needle, with a diameter of 2.12 mm, weighed 113 g, while the final setting needle, with a diameter of 1.06 mm, weighed 453.6 g. To assess the setting times, cementitious pastes were placed in a specialized form (8 mm × 10 mm × 5 mm), and the needle was gently applied to its surface. The setting time was determined as the moment when the needle did not leave a distinct circular mark on the surface. All experiments were conducted at room temperature (22 ± 1 °C). The results were shown as the average of three measurements and accompanied by their respective standard deviations (SD).

#### 2.2.3. Mechanical Strength

To assess the compressive strength of the materials, cylindrical samples with a height of 12 mm and a diameter of 6 mm underwent testing using a universal material testing machine (Instron 3345, Norwood, MA, USA) at a crosshead displacement rate of 1 mm·min⁻^1^. The results for compressive strength were reported as the mean value ± the standard deviation (SD) based on twenty determinations. Statistical analysis to identify any significant differences between the materials involved a one-way ANOVA followed by post hoc Tukey’s Honest Significant Difference (HSD) test with a significance level of *p* < 0.05.

#### 2.2.4. Microstructure

To examine the microstructure of the fractured samples, scanning electron microscopy (SEM) was applied with a PhenomPure instrument from Thermo Fisher Scientific, located in Waltham, MA, USA. To assess the bioactive property, the surfaces of the biomaterials underwent examination after 7 days of incubation in simulated body fluid (SBF) at 37 °C. Before analysis, a thin layer of gold film was applied to the samples. Employing a low deposition rate was crucial to prevent the accumulation of charges and improve the resolution of imaging.

#### 2.2.5. In Vitro Bioactivity and Chemical Stability

To evaluate the chemical stability and bioactivity of the bone cements, they were incubated in distilled water or simulated body fluid (SBF) prepared according to Kokubo’s protocol [20]. Cylindrical samples were placed in containers containing 50 mL of simulated body fluid (SBF) or water and were stored at 37 °C. The chemical stability of the materials was evaluated by monitoring the pH and ionic conductivity of the solutions surrounding the incubated samples over different time intervals. These measurements were conducted using a Seven Compact Duo pH/conductometer (Mettler Toledo, Columbus, OH, USA). The results were presented as the mean of three measurements ± standard deviation (SD). After immersion, the samples were taken out from the solution, washed with distilled water, and dried at temperatures lower than 40 °C. To confirm their bioactive potential, the surfaces of the samples were analyzed using scanning electron microscopy (SEM).

#### 2.2.6. Evaluation of Cytotoxicity

The in vitro cytotoxicity of the microparticles was evaluated according to ISO 10993-5 [21] in extracts of the materials in the culture media. For this approach, mouse fibroblasts L929 (CCL-1, American Type Culture Collection) were used. The cells were cultured in Dulbecco’s modified Eagle’s medium (DMEM, PAN-Biotech, Aidenbach, Germany) supplemented with 10% fetal bovine serum (FBS, South America origin, PAN-Biotech, Aidenbach, Germany) and 1% penicillin/streptomycin (PAN-Biotech). The cell culture was performed in a humidified atmosphere at 37 °C with 5% CO_2_. The extracts were prepared by incubation of the materials in the culture medium at 37 °C for 24 h at the concentration of 10% (100 mg/mL), and they were later 3 times diluted twofold to obtain the concentrations of 5%, 2.5%, and 1.25%, respectively. The sterility of the extracts was assured by filtration through 0.2 µm filters.

The cells were seeded in 96-well plates at 10,000 cells/well in 100 μL of medium and were cultured for 24 h to allow adhesion to the well bottom and initial proliferation. Then, the cell culture medium was replaced with 100 μL of the extracts at the above-mentioned concentrations. The experiment was run in triplicate for each sample, and the concentration was tested. After 24 h of incubation, the cell viability was evaluated using a resazurin reduction assay and live/dead staining.

##### Evaluation of Cytotoxicity

A resazurin reduction assay was applied to assess the metabolic activity of the cells. The extracts were removed, and wells with cells were rinsed with PBS. Next, to each well with cells, 150 μL of Alamar Blue reagent (resazurin sodium salt dissolved in PBS at 0.1 mg/mL concentration and then incorporated into the medium at a concentration of 10% (*v*/*v*)) was added and incubated for 3 h at 37 °C. Then, 100 μL of post incubation media with Alamar Blue reagent was transferred into a black 96-well plate. The fluorescence was measured at λex = 544 nm, λem = 590 nm via a microplate reader (FluoroSTAR Omega, BMG Labtech, Ortenberg, Germany). The percentage of resazurin reduction was calculated based on Equation (1) and compared to the control sample (cells incubated in pure medium).

(1) $$RR=\frac{Fluo_{x}-Fluo_{0}}{Fluo_{100}-Fluo_{0}}$$

where RR—resazurin reduction, Fluo_x_—fluorescence of the sample, Fluo_0_—fluorescence of the background (AlamarBlue reagent in culture medium incubated in empty wells), Fluo_100_—fluorescence of the AlamarBlue reagent in culture medium reduced completely in an autoclave (121 °C, 15 min).

##### Live/Dead Fluorescent Staining

Live/dead staining was performed to evaluate the cell viability and morphology. The extracts were removed, and the cells were rinsed with PBS. Then, the PBS solution containing 0.1% calcein AM (Sigma-Aldrich, Steinheim, Germany, CAS:148504–34-1) and 0.1% propidium iodide (Sigma-Aldrich, CAS:25535–16-4) was added to the wells. The cells were incubated with the dyes (20 min in the dark, 37 °C). Fluorescent microscopy images were taken using a fluorescence microscope (Axiovert 40 CFL with HXP 120C Metal Halide Illuminator, Zeiss, Oberkochen, Germany) at 100× magnification.

## 3. Results and Discussion

### 3.1. Phase Composition

**Figure 2:**
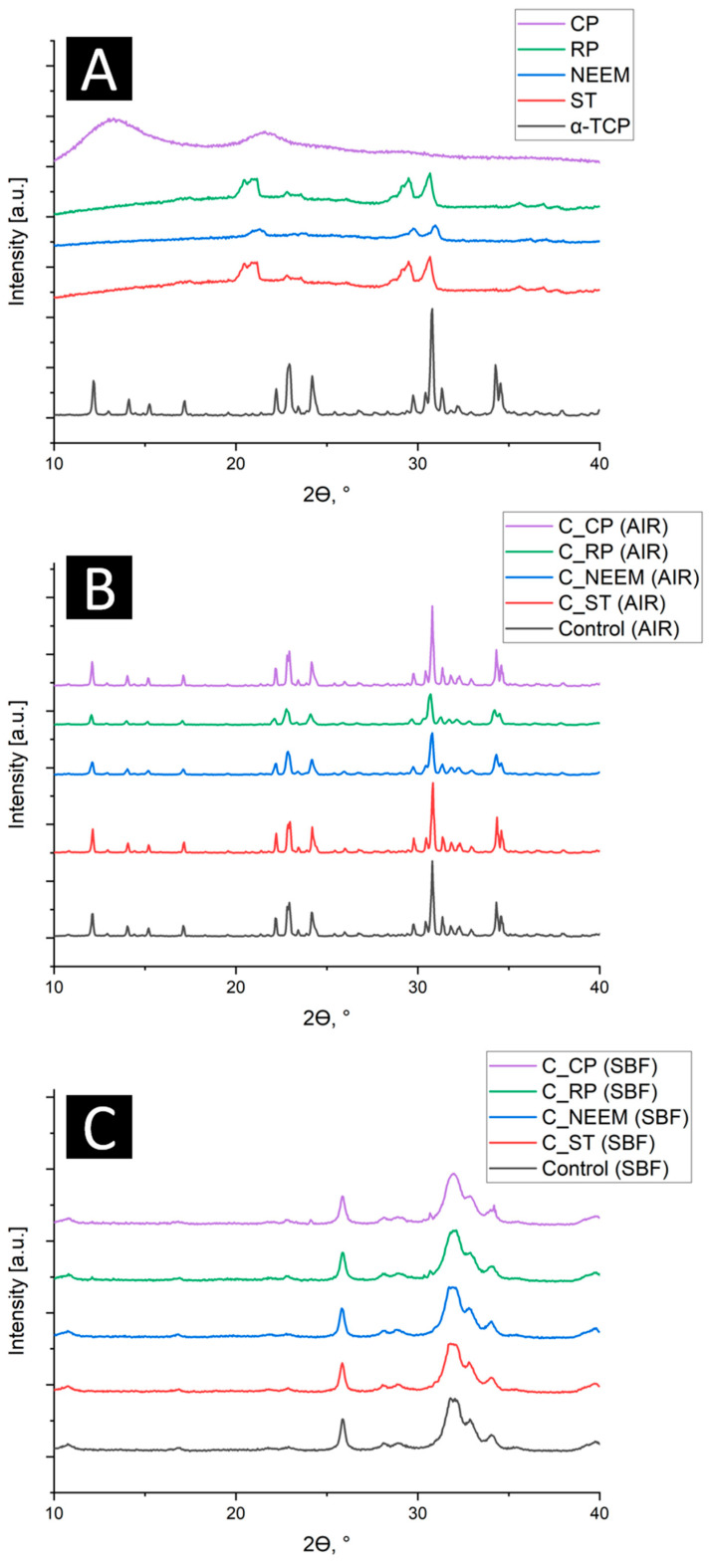
XRD patterns of studied materials: initial powders (**A**), after setting and hardening in air (**B**), and after incubation in SBF of 7 days (**C**).

Quantitative X-ray diffraction analysis revealed that the initial α-TCP powder was composed of the α-TCP phase (98 ± 1 wt.%) and a small amount of hydroxyapatite (2 ± 1 wt.%). The XRD pattern of citrus pectin exhibits an amorphous halo, typical for polymeric materials, whereas synthesized PAAM modified with sago starch (ST), neem flower (NEEM), and rambutan peel (RP) possessed a semi-crystalline structure with the presence of reflexes characteristic for crystalline materials. In bone cements, XRD measurement confirmed the presence of two crystalline phases: α-TCP and hydroxyapatite (Figure 2). Due to the small amount of polymeric additives in cementitious materials, the amorphous halos originating from polymers were not visible in the cements’ diffractograms. The crystalline reflexes of reinforced PAAM overlap with reflexes from calcium phosphates (α-TCP and hydroxyapatite).

The quantitative phase composition of obtained modified bone cements is presented in Table 2.

The amount of α-TCP phase in the materials after 7 days of setting and hardening in the air ranged from 86 ± 1 wt.% to 90 ± 1 wt.%, while the amount of hydroxyapatite phase ranged from 10 ± 1 wt.% to 14 ± 1 wt.%. The minor deceleration in α-TCP hydrolysis to hydroxyapatite can be explained by the fact that the polymer additives in the form of hydrogel absorbs water molecules and hinders this process [22,23]. Phase composition analysis of the materials after a week of incubation in simulated body fluid (SBF) revealed that the α-TCP phase in the aqueous environment spontaneously hydrolyzes into hydroxyapatite with a calcium deficit. Similar observations were previously noted for complex cementitious systems previously [24,25].

### 3.2. Setting Times

In clinical applications, cementitious bone substitutes need to exhibit setting times that facilitate their application by surgeons to the specific defect site. Ideally, the initial setting time (ti) should be within the range of 4–8 min, and the final setting time (tf) should not exceed 15 min [25].The setting times of bone cements depended on the type of used polymeric additive and varied between 2.5 and 12.5 min (initial setting time) and 8.0 and 21.0 (final setting time) (Table 3).

The reaction of α-TCP with water results in the formation of calcium-deficient apatite, as expressed by the following Equation (2) [26,27]:

α-3Ca_3_(PO_4_)_2_ + H_2_O → Ca_9_(PO_4_)_5_(HPO_4_)OH (2)

α-TCP + H_2_O → non-stoichiometric hydroxyapatite

The setting process may be influenced by the following factors: (1) the quantity of the reactive α-TCP phase, (2) the ratio of the powder to liquid phase (L/P), (3) the presence of a setting reaction accelerator in the liquid phase (e.g., Na_2_HPO_4_), as well as the presence of other components in the material, both in the powder and liquid phases [28]. Usually, the addition of polymers to bone cements results in an extended setting time, as observed in the case of material with citrus pectin (C_CP). However, in the case materials containing polyacrylamide modified with various natural origin substances (Sago Starch, Neem flower, Rambutan peel), shortened setting times were observed. These results demonstrate that the setting properties of α-TCP-based paste is highly dependent on the physicochemical properties of polymeric additives—primarily their behavior during the mixing with the liquid phase. Certain polymers undergo dissolution in the aqueous environment; some swell by absorbing a substantial amount of water, while others remain nearly inert upon contact with water. For the applied polymers, various mechanisms were observed. In the case of materials, the shortened setting times of C_ST, C_NEEM, and C_RP seem to be due to water absorption and the swelling of the modified PAAM after mixing with the liquid phase of the cements. Afterward, the PAAM may release a portion of water molecules into the system. Whereas, for material C_CP (with citrus pectin), the elongation of the setting process is associated with water uptake by citrus pectin followed by polymer dissolution [29,30]. The comparable interval (~8 min) between the initial and final setting times for both the control and C_CP suggests that the extended setting time is likely linked to a delay in the initial stages of the α-TCP setting process. This delay is attributed to the citrus pectin hindering water access to α-TCP. In this case, dissolved citrus pectin covers α-TCP grains and creates a barrier for water molecules. The presence of this barrier decelerate the setting of α-TCP. Simultaneously, in the presence of Ca^2+^ ions, a gelation occurs, and higher levels of calcium ions may lead to syneresis (expulsion of a liquid from a gel) as well as the creation of an insoluble complex and phase separation [31].

### 3.3. Mechanical Strength

**Figure 3:**
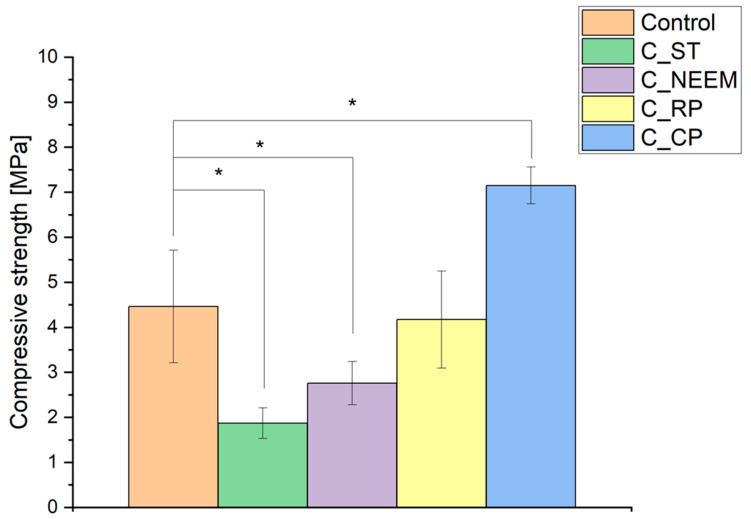
Compressive strength of bone cements after 7 days of setting and hardening in air (statistically significant difference at * *p* < 0.05).

The mechanical strength of calcium phosphate-based bone cements holds significant importance, as it dictates the cement’s capacity to withstand the mechanical forces in the bone environment, ensuring effective bone healing [32].The results of the compressive strength tests of obtained materials are shown in Figure 3.

The compressive strength of the control cement material was 4.46 ± 1.25 MPa. In the case of bone cements modified with PAAM, a decrease in mechanical properties was observed. The compressive strength was between 1.87 ± 0.34 (C_ST) and 4.17 ± 1.08 MPa(C_RP). The modification of the biomaterial composition with citrus pectin resulted in an increase in the material’s compressive strength to 7.15 ± 0.41 MPa (C_CP).

The main reason for the compressive strength decrease is the non-homogenous microstructure of cementitious materials containing modified PAAM. The differences in the mechanical strength of materials may also be attributed to their modification with sago starch, neem flower, or rambutan peel and various behaviors during the setting process [33]. In the case of citrus pectin, the enhancement of compressive strength could occur in a dual manner. The first is via ensuring a better homogenization of cementitious pastes through the presence of citrus pectin. Citrus pectin acts as a well-soluble hydrogel, allowing for its homogeneous distribution in the cement paste. Additionally, pectin causes the thickening of the cementitious paste during its formation. The second reason for the increase in mechanical strength is due to the pectin cross-linking by Ca^2+^ ions [34]. The cross-linking process is believed to follow to the egg-box model in which calcium ions fit the junction zones and bind the pectin chains together via electrostatic and ionic interaction [31]. Thus, the improved mechanical properties can be attributed to the denser microstructure and the increased bonding strength within the cementitious matrix. A similar favorable impact of adding citrus pectin on the strength of materials based on calcium phosphates has been previously observed [35,36]. The mechanical strength of the developed materials enables their implantation in non-load or low-load-bearing locations, considering that the compressive strength of trabecular bone is from 0.1 to 16 MPa [37].

### 3.4. Microstructure

To assess the suitability of the developed materials as substitutes for bone tissue and identify any microstructural defects, their microstructure underwent examination. The obtained biomaterials were observed using scanning electron microscopy (SEM) both after 7 days of setting and hardening in air as well as after 7 days of sample incubation in SBF.

**Figure 4:**
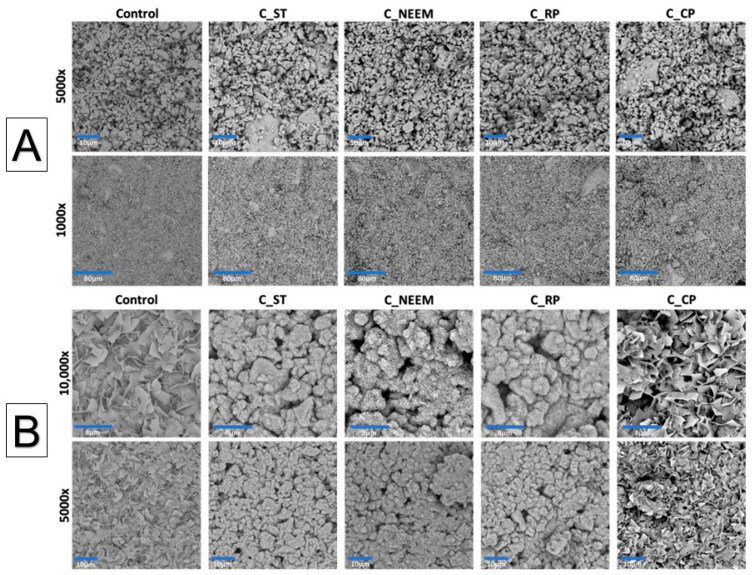
SEM microphotographs of the materials’ cross-section after 7 days in air (**A**) and after 7 days incubation in SBF (**B**).

The observed cementitious samples after 7 days of setting and hardening in air possessed a compact and homogeneous microstructure formed by an α-TCP-based cementitious matrix with visible micropores (Figure 4). Importantly, no visible differences were observed between the control material and materials in which polymeric modifiers were added. Similar microstructures of α-TCP-based bone cements can be found in other studies [38].

After 7 days of incubation in simulated body fluid (SBF), the surfaces of all observed biomaterials were completely covered by a plate-like apatitic structure, confirming their high bioactive potential in vitro according to Kokubo and Takadama’s protocol (Figure 4B) [39]. Moreover, the microstructural analysis post-incubation demonstrated that the developed materials maintained their bioactive potential similar to α-TCP-based bone cements despite using different polymeric additives.

### 3.5. Chemical Stability and Bioactivity

**Figure 5:**
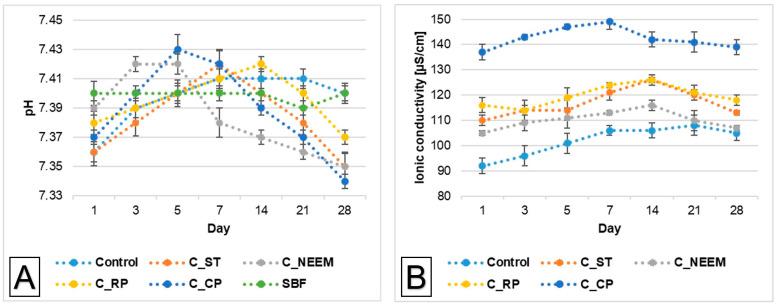
pH values versus time of incubation of obtained bone cements in SBF (**A**) and ionic conductivity values versus time of incubation in distilled water (**B**).

The essential factor for assessing the potential clinical use of implantable biomaterials is their chemical stability. Figure 5A illustrates the pH changes in the simulated body fluid (SBF) during the sample’s immersion.

The pH changes of SBF around incubated samples remained close to the physiological values and ranged from 7.34 to 7.43 (Figure 5B). The addition of polymeric additives only slightly influenced the solution’s pH values. Comparable pH values were noted in other studies involving incubated calcium phosphate-based bone substitutes [40].

Ionic conductivity during the incubation in distilled water of the control material was in the range of 92–106 μS/cm. Polymeric modifiers caused a slight increase in the ionic conductivity to the range of 110–120 μS/cm for sago starch-modified PAAM, to the range of 105–116 μS/cm for neem flower-reinforced PAAM, and to the range of 114–126 μS/cm for rambutan peel-reinforced PAAM. The highest ionic conductivity was observed for materials in which citrus pectin was used (137–149 μS/cm) (Figure 4B). This phenomenon can be explained by a higher degradation rate of polymeric additives, which all are soluble in water [41,42,43].

The ionic conductivity of all the obtained materials did not stand out significantly compared to the ionic conductivity of α-TCP-based cementitious materials. It can therefore be concluded that the modification of calcium phosphate-based bone cements with different polymers did not significantly alter their degradation rate.

### 3.6. Evaluation of Cytotoxicity

**Figure 6:**
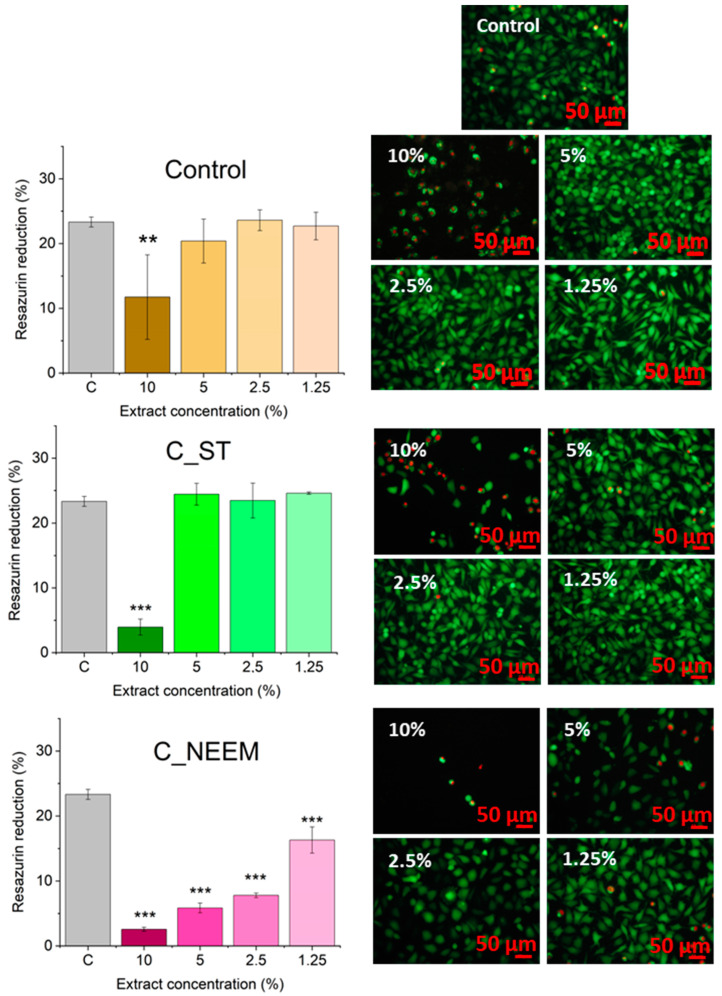

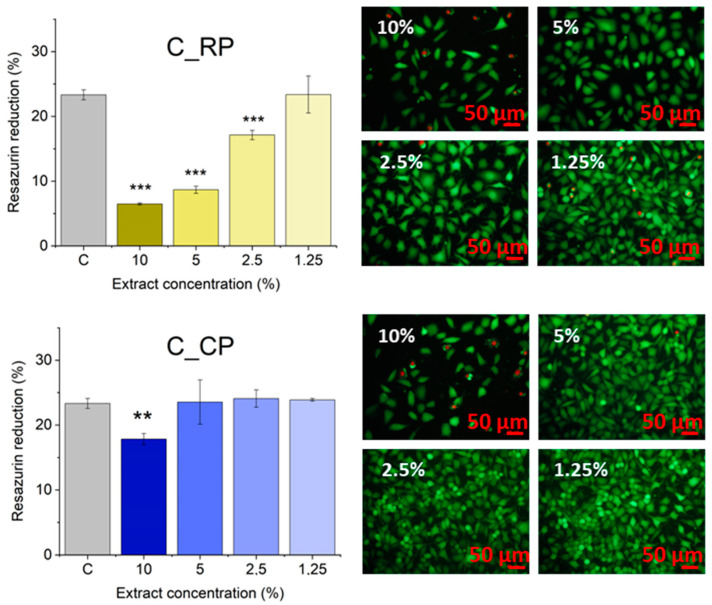
Cytotoxicity tests: Alamar Blue metabolic assay (**left** panel), ** *p* < 0.01, *** *p* < 0.001, and live/dead fluorescent staining (**right** panel) of extracts from the respective cement samples.

The cytotoxicity experiments included tests of metabolic activity based on resazurin reduction (AlamarBlue) and fluorescent live/dead staining (Figure 6). All 10% extracts of the cement samples significantly reduced L929 cell viability as compared to cells cultured in control conditions, i.e., DMEM. At this concentration, some living cells (stained green) could be found in all samples, while most of them were dead (stained red). According to ISO 10993-5, materials are considered cytotoxic if the viability reduction is higher than 30%. Taking this standard into account, C-CP is not cytotoxic, even at the highest 10% concentration of the extract. In this sample, the highest number of live cells was also observed. C_NEEM and C_RP showed higher cytotoxicity as their 1.25% and 2.5% extracts significantly reduced the cells’ viability, respectively. Control cement, as well as C_ST and C_CP samples, showed no decrease in metabolic activity for concentrations of 5% and lower. Also, there were no differences between those samples and the reference cells incubated in pure medium by fluorescence microscopy observations. The C_CP and C_ST samples’ cytocompatibility was therefore not affected by the introduced modifications.

Although the 10% extracts were toxic for all the materials, this concentration is really high. They were obtained by incubating 100 mg of the polymer per just 1 mL of DMEM. Nabavizadeh et al. [44] tested the cytotoxicity of their bone cements with L929 cells as well. They obtained the most concentrated extracts by incubating just 1 mg of the material in 5 mL of DMEM. Still, their extracts were toxic up to 1:16 dilution. We assume that only the C_NEEM and C_RP samples were indeed toxic for even the low concentration, and their potential in bone tissue engineering might be limited as a result. The other materials should not be toxic, as even the currently used glass ionomer cement can affect L929 cells significantly at 5% (50 mg/mL) [45]. C_CP might be even less toxic than the control material; however, it requires further confirmation due to the wide distribution of the results for the control (10% extracts).

## 4. Conclusions

In this study, materials based on highly reactive α-TCP powder were developed and examined. The effect of PAAM supplemented with different plant-based additives to the powder phase of developed cement materials was assessed. The presence of citrus pectin significantly improved the mechanical strength of the resulting materials (from 4.46 to 7.15 MPa). The improved mechanical properties of this type of cement resulted from the better homogenization of cementitious pastes as well as pectin cross-linking by Ca^2+^ ions. The acceptable elongation of the setting process was also observed for this material. In the case of cements with PAAM modified with sago starch, neem flower, and rambutan peel, a decrease in the mechanical strength and setting times was observed. All the developed bone cements revealed in vitro bioactivity according to the SBF test. Cement modified with pectin was found to be cytocompatible according to ISO 10993-5 for all extract concentrations. Materials containing sago starch were more cytocompatible than those supplemented with neem flower and rambutan peel, making these two modifiers less promising candidates for bone tissue engineering. The material modified with citrus pectin is considered to be the most promising among the materials studied. Further biological experiments are necessary to confirm this hypothesis.

## Figures and Tables

**Table 1:** Initial composition of developed materials.

Label	Powder Phase	Liquid Phase	L/P [g/g]
Control	α-TCP	2 wt.% Na_2_HPO_4_ solution in distilled water	0.45
C_ST	α-TCP + 3.0 wt.% sago starch modified PAAM		
C_NEEM	α-TCP + 3.0 wt.% neem flower modified PAAM		
C_RP	α-TCP + 3.0 wt.% rambutan peel modified PAAM		
C_CP	α-TCP + 3.0 wt.% citrus pectin		

**Table 2:** Phase composition of set and hardened cements after 7 days in air or SBF.

Material	7 Days in Air		7 Days in SBF	
	α-TCP, wt.%	Hydroxyapatite, wt.%	α-TCP, wt.%	Hydroxyapatite, wt.%
Control	86 ± 1	14 ± 1	2 ± 1	98 ± 1
C_ST	90 ± 1	10 ± 1	5 ± 1	95 ± 1
C_NEEM	88 ± 1	12 ± 1	4 ± 1	96 ± 1
C_RP	86 ± 1	14 ± 1	4 ± 1	96 ± 1
C_CP	88 ± 1	12 ± 1	3 ± 1	97 ± 1

**Table 3:** Setting time of obtained bone cements.

Material	Initial Setting Time, min	Final Setting Time, min
Control	6.0 ± 0.5	14.0 ± 1.0
C_ST	2.5 ± 1.0	10.0 ± 1.5
C_NEEM	3.0 ± 1.0	11.5 ± 0.5
C_RP	3.0 ± 0.5	8.0 ± 0.5
C_CP	12.5 ± 1.0	21.0 ± 1.0

## Data Availability

The original contributions presented in the study are included in the article; further inquiries can be directed to the corresponding authors.
